# 189. Risk Factors for the Progression from Clostridioides difficile Colonization to Infection: A Single-Center, Retrospective Case-Control Study

**DOI:** 10.1093/ofid/ofaf695.064

**Published:** 2026-01-11

**Authors:** Sophia Chang, Deverick J Anderson, Michael E Yarrington, Nicholas A Turner

**Affiliations:** Duke University School of Medicine, Durham, NC; Duke Center for Antimicrobial Stewardship and Infection Prevention, Durham, NC; Duke University Health System, Durham, North Carolina; Duke University Medical Center, Durham, NC

## Abstract

**Background:**

*Clostridioides difficile* infection (CDI) remains the leading cause of healthcare–associated infection. Patients colonized with toxigenic *C. difficile* are at an increased risk of developing CDI. Data remains limited on the distinct host and clinical characteristics that impact this risk of progression.

**Figure:**
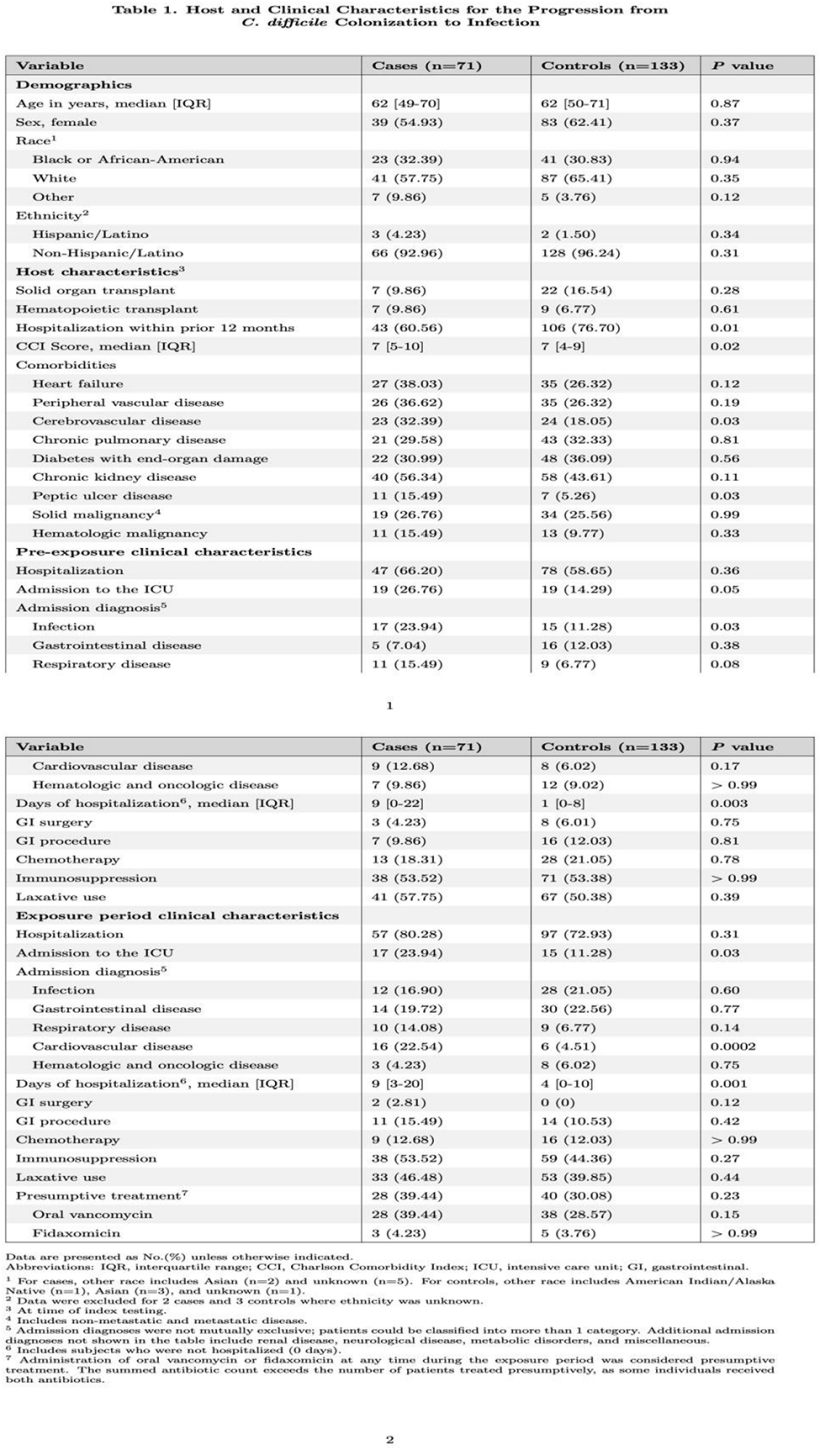

**Figure:**
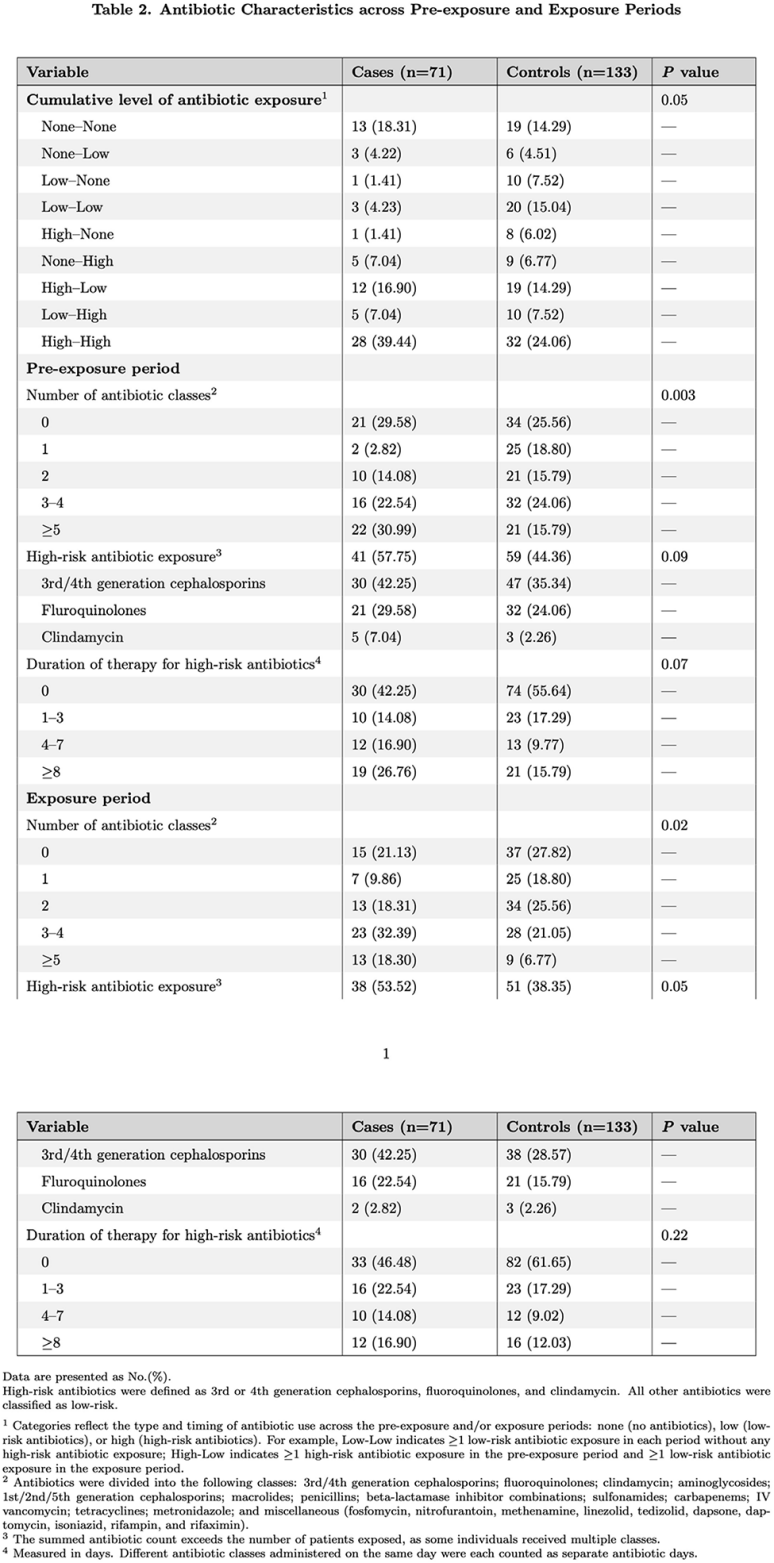

**Methods:**

We conducted a retrospective matched case-control study of adult patients (≥18 years) who underwent >1 two-step stool test for *C. difficile* within the Duke University Health System between 03/15/2020-12/31/2023. Cases were patients with *C. difficile* colonization (NAAT+/toxin-) who progressed to CDI (NAAT+/toxin+) within 90 days; controls were colonized patients who remained toxin-negative. Cases were matched to controls based on date of index testing (±1 year). Data collection included host/clinical characteristics during a 90-day “pre-exposure” period preceding index testing and a ≤90-day “exposure” period between index and repeat testing. Antibiotic exposure was stratified into risk categories— high-risk, low-risk, or none—for each period. Multivariable conditional logistic regression with forward selection was used to identify independent variables associated with progression to CDI.

**Figure:**
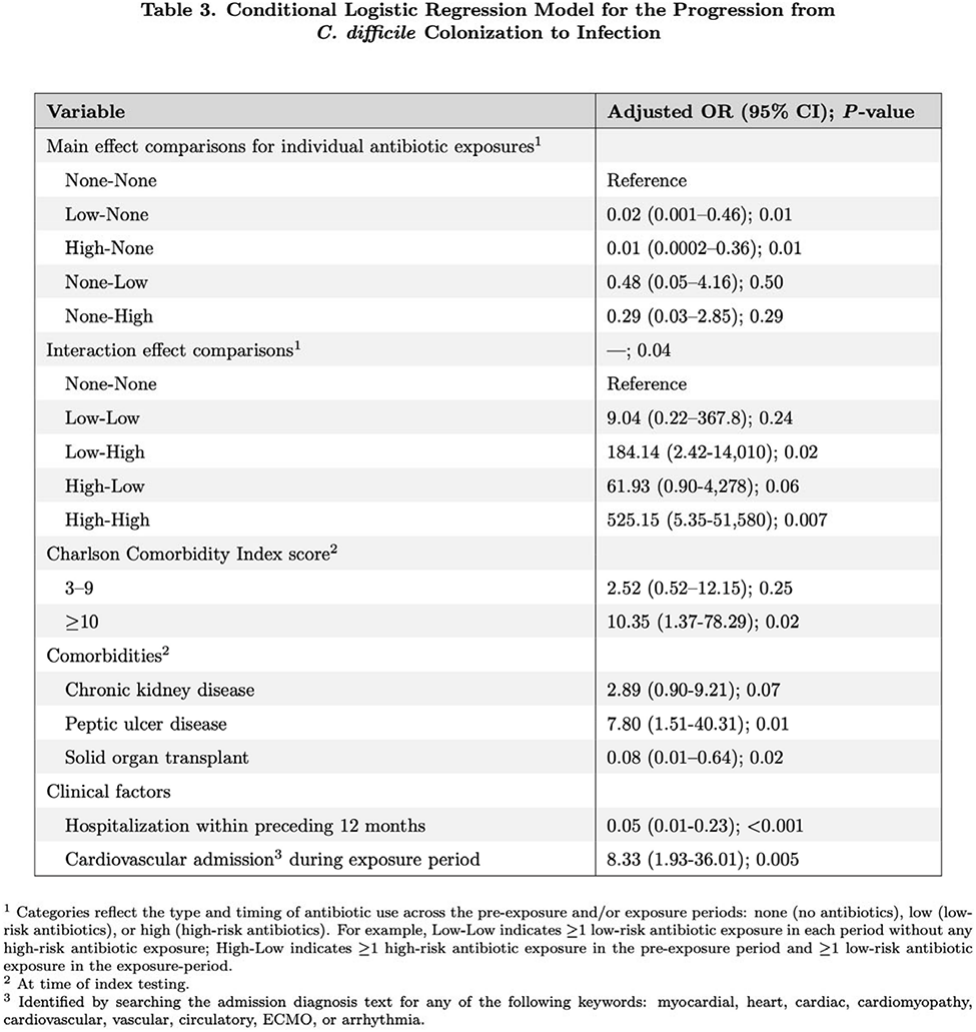

**Results:**

Among 2,212 individuals with *C. difficile* colonization, we identified 71 cases and 133 matched controls. Host and clinical characteristics are summarized in Table 1. Antibiotic data are detailed in Table 2. Several variables were independently associated with progression to CDI in our multivariable model (Table 3). An interaction term combining pre-exposure and exposure period antibiotic use was statistically significant (p=0.04). In particular, receipt of high-risk antibiotics in both the pre-exposure and exposure period was highly predictive of progression via evaluation of the interaction term (aOR 525.15, 95% CI (5.35-51,580); p=0.007).

**Conclusion:**

Sustained exposure to high-risk antibiotics was associated with progression from *C. difficile* colonization to infection. These findings highlight the critical importance of antibiotic stewardship not only in the period following identification of colonization but also in preceding healthcare exposures. Further studies are needed to determine the best strategies for CDI prevention and management.

**Disclosures:**

Nicholas A. Turner, MD, MHSc, Basilea: Clinical trial adjudicator for ceftobiprole|PDI: Grant/Research Support|Purio Labs: Grant/Research Support

